# Effectiveness of professional and patient-oriented strategies in reducing vitamin D and B12 test ordering in primary care: a cluster randomised intervention study

**DOI:** 10.3399/BJGPO.2021.0113

**Published:** 2021-10-27

**Authors:** Saskia van Vugt, Evelien de Schepper, Sanne van Delft, Nicolaas Zuithoff, Niek de Wit, Patrick Bindels

**Affiliations:** 1 Department of General Practice, Julius Center for Health Sciences and Primary Care, University Medical Center Utrecht, Utrecht, The Netherlands; 2 Department of General Practice, Erasmus Medical Center, Rotterdam, The Netherlands; 3 Saltro, Diagnostic Center for Primary Care, Utrecht, The Netherlands

**Keywords:** overdiagnosis, primary care, vitamins, diagnostic tests, routine, Netherlands, vitamin B12, vitamin D

## Abstract

**Background:**

Vitamin tests are increasingly ordered by GPs, but a clinical and evidence-based indication is often lacking. Harnessing technology (that is, decision support tools and redesigning request forms) have been shown to reduce vitamin requests.

**Aim:**

To investigate whether the number of vitamin tests may be reduced by providing a multi-level intervention programme based on training, monitoring, and feedback.

**Design & setting:**

This was a cluster randomised intervention study performed in 26 primary care health centres (>195 000 patients) in the Netherlands. The relative reduction in ordered vitamin D and B12 tests was determined after introduction of two de-implementation strategies (1 May 2017 to 30 April 2018).

**Method:**

Health centres randomised to de-implementation strategy 1 received education and benchmarking of their own vitamin test ordering behaviour every 3 months. Health centres in de-implementation strategy 2 received the same education and benchmarking, but supplemented with educational material for patients.

**Results:**

The number of vitamin D tests decreased by 23% compared to the 1-year pre-intervention period (1 May 2016 to 30 April 2017). For vitamin B12 tests an overall reduction of 20% was found. Provision of patient educational information showed additional value over training and benchmarking of GPs alone for vitamin D test ordering (10% extra reduction, odds ratio [OR] 0.88, 95% confidence interval [CI] = 0.83 to 0.92), but not for vitamin B12 ordering (4% extra reduction, OR 0.96, 95% CI = 0.91 to 1.02). Nationwide, this would result in over €3 200 000 in savings on healthcare expenditure a year.

**Conclusion:**

A structured intervention programme, including training and benchmarking of GPs regarding their diagnostic test ordering, resulted in a significant reduction in ordered vitamin tests. Additional information provision to patients resulted in a small but still relevant additional reduction. If implemented on a national level, a substantial cost saving could be achieved.

## How this fits in

Vitamin tests are increasingly ordered by GPs, but a clinical and evidence-based indication is often lacking. This cluster randomised intervention study showed that with a structured time-limited intervention programme, including training and benchmarking of GPs, a significant reduction in the number of vitamin tests in primary care can be achieved with substantial cost saving.

## Introduction

Medical overuse, including both overdiagnosis and overtreatment, is a growing problem in health care.^1^ Overuse is increasingly recognised around the world, but quantifying it is often challenging. Estimates of costs related to overuse vary widely, but overuse of individual services may occur in as many as 80% of cases.^2^

Despite the growing awareness and recommendations from the Choosing Wisely campaign (https://www.choosingwisely.org), several studies illustrate how difficult it is to achieve substantial reduction in unnecessary testing.^3–5^ A recent UK study showed that diagnostic testing in primary care substantially increased in the period from 2000–2015.^5^ Testing for vitamin D increased exponentially, with an average annual increase of 54%. Also, a linear increase pattern emerged for vitamin B12, with an annual increase of 17%.^5^ Although recommended for specific patient populations,^6,7^ laboratory tests for vitamins D and B12 are mainly used for patients with non-specific symptoms.^8,9^

Although many consider vitamin testing to be ‘harmless’, it may lead to medicalisation (owing to untargeted testing in response to [irrational] health perceptions of patients) or overdiagnosis (‘iatrogenic illness’),^10^ and thereby to an unnecessary increase of healthcare expenditure.^5,11^ This highlights the need to rationalise vitamin test ordering — especially vitamins D and B12, the most frequently ordered vitamin tests in clinical practice — through influencing both medical professionals and patients.^12^

A systematic review showed how involving patients through patient-targeted educational materials is effective in decreasing the use of low-value care.^12,13^ Furthermore, previous studies showed how redesign of the electronic request form^14^ and three clinical decision support tools (guideline development, a ‘stop alert’ shown to the ordering clinician, and removal from the laboratory ordering preference list)^15^ reduced vitamin D testing by 36% and 30%, respectively. However, the individual impact of these tools could not be assessed, the follow-up period was limited to 6 months, and implementation is only possible in a setting where decision support tools can be integrated into electronic health records.^13–15^ Therefore, this study focused on other simple intervention strategies that can be implemented in every primary care system and be evaluated separately. In this study, therefore, the effect of a GP-targeted intervention programme based on training, monitoring, and feedback to rationalise vitamin D and B12 test ordering in primary care was assessed, as was the added value of practice-based patient information about vitamins and health.

## Method

### Design

A cluster randomised intervention study comparing two de-implementation strategies.

### Setting

Health centres from two regional academic primary care networks in the Netherlands were invited to participate in a 1-year intervention study. All vitamin D and B12 tests requested by GPs working in the 26 health centres during the intervention year (1 May 2017 to 30 April 2018) and the pre-intervention year (1 May 2016 to 30 April 2017) were extracted.

### Participants

All 59 health centres from the Julius General Practitioners’ Network (JGPN) Utrecht,^16^ two health centres of the Academic Primeur network, and eight other health centres in the Rotterdam area were invited to participate.

Criteria for inclusion were willingness to attend two obligatory educational sessions by at least one GP of the participating centre, and permission to extract data from the regional diagnostic laboratory on their vitamin D and B12 test ordering. There were no exclusion criteria.

### Interventions

**Figure 1.**
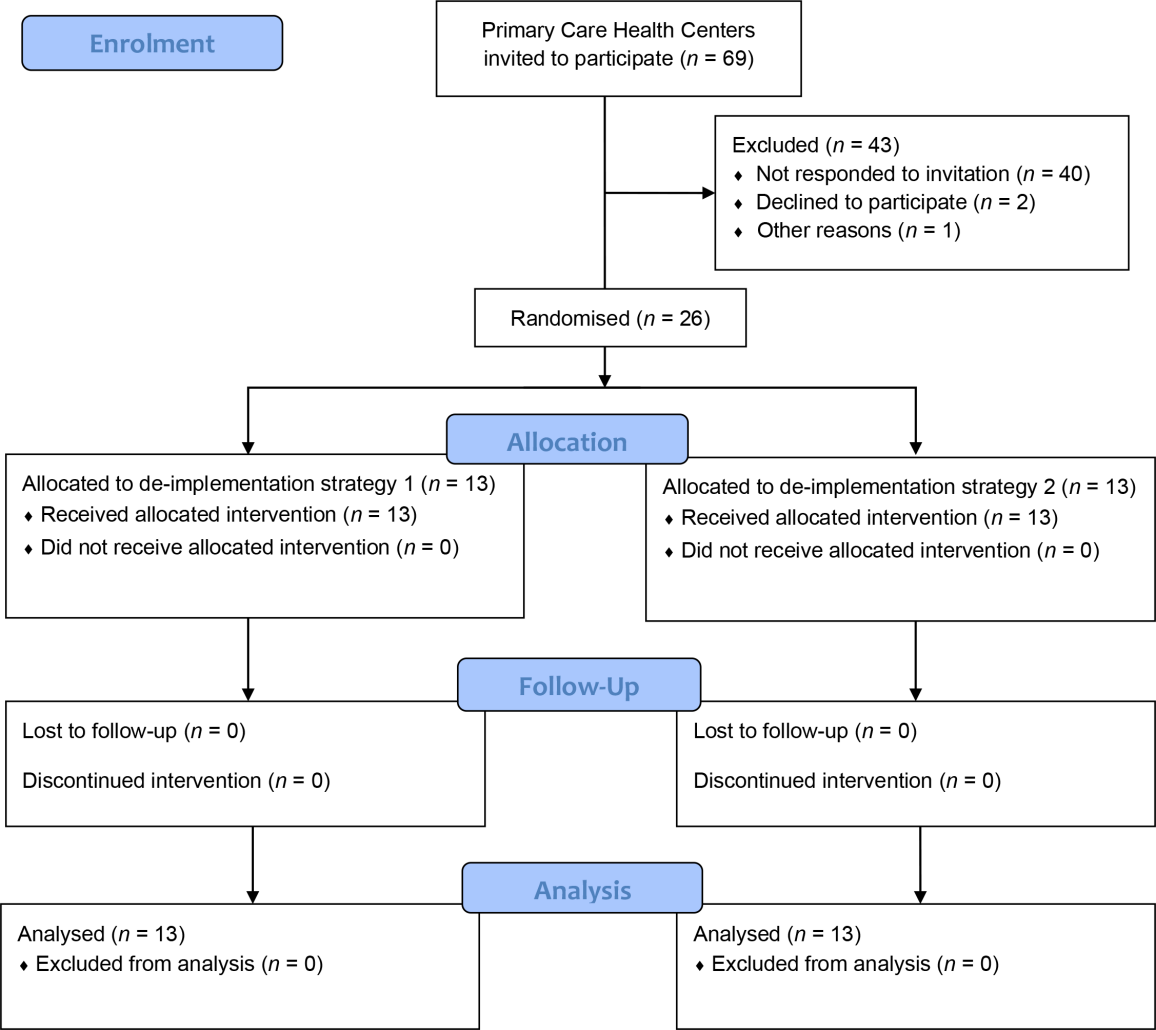
Flow diagram of the study and its participating health centres

The participating health centres were randomised to either de-implementation strategy 1 (‘GP only’) or strategy 2 (‘GP and patient’) (Figure 1).

Strategy 1 included two training sessions of 1.5 hours, the first session addressed the evidence and indications for vitamin D and B12 testing, the second covered communication strategies regarding (withstanding) patients’ requests for testing. At least one GP from each centre had to attend the plenary meeting. All other GPs were allowed to follow the e-learning of this educational session. GPs received emails every 3 months containing the number of ordered vitamin tests, benchmarked to the numbers of the other participating health centres.

In strategy 2, the health centres received the same training, monitoring, and feedback as in strategy 1, but in addition, these centres were equipped with educational material for patients through video screens in the waiting room, as well as leaflets in the Dutch, Turkish, and Arabic languages about the health effects of vitamins.

### Outcomes and measurements

The primary outcome was the difference in the number of vitamin D and B12 blood tests ordered by GPs during the intervention year controlled for the number in the pre-intervention year.

Secondary outcomes were the number of abnormal test results and the direct cost savings.

Data on pre-intervention and intervention vitamin testing were collected through the primary care laboratory organisations in Utrecht (Saltro) and Rotterdam (Star-shl). Both organisations have very long-standing relations with their regional GPs, handling >90% of the laboratory tests ordered by GPs in their region. GP and health-centre identity (code), age and sex of patient, date, test (for example, vitamin D or B12), and test result were extracted anonymously from existing registries.

### Randomisation

Randomisation was performed by the Data Management Department of the Julius Centre for Health Sciences and Primary Care Utrecht and controlled for region (Utrecht/Rotterdam) and health-centre size to ensure proper distribution over both intervention arms. The intervention allocation within this cluster randomised intervention study could not be blinded.

### Sample size calculation

Based on a two-sample Wilcoxon sample size calculation with data on the achieved absolute reduction in vitamin D tests in an earlier performed ‘practice improving project’ in the Rotterdam region (that is, Poisson means of 850 tests before and 638 after intervention), an alpha of 0.05, power of 0.90, and a minimum number of 36 individual GPs (with a mean of 2095 patients each) were required to achieve significance. When taking an expected cluster correlation of 0.15 into account, the number of individual GPs required increased to 66. Allowing for drop-outs, this study aimed to include 75 individual GPs, with a resulting patient population of approximately 157 125.

### Data analysis

The primary outcome (difference in the number of requested tests [divided by the number of patients] in pre- and post-intervention year per health centre) was analysed with a generalised linear mixed model for binomial outcomes. A random intercept was included to correct for clustering (owing to the repeated measurement in each centre). The comparison between strategy 1 and 2 during the intervention year was included separately. In an additional step, predefined confounders were included; that is, the number and sex of GPs per health centre, and (to correct for composition of the patient population)^17^ the average socioeconomic status (SES) of patients in each centre. SES data were retrieved from the Social and Cultural Planning Office (SCP), which calculates SES scores based on information regarding education, income, and position in the labour market.^18^

The effects of the intervention strategies were reported in ORs with 95% CIs and *P*-values.

To externally validate the results, comparison with the number of ordered vitamin tests in the same period by non-participating health centres was performed. These (anonymous) data were retrieved from Saltro’s laboratory registry, which contains routine data of all tests requested by primary care health centres in the Utrecht region.

Presuming that the average test result is an adequate proxy for the quality of the indication of a performed test, the number of tests indicating a vitamin deficiency was determined (based on reference values from the Dutch GP guidelines on vitamin D^19^ and B12^20^).

Finally, direct cost savings were determined by calculating savings from the reduction in the number of vitamin D and B12 tests. Standard national tariffs for vitamin D and B12 laboratory tests were used;^21^ that is, €7.62 to €8.38 (£6.54 to £7.20) (average €8.00 [£6.87]) for vitamin D, and €5.81 to €6.39 (£4.99 to £5.49) (average €6.10 [£5.24]) for vitamin B12. Other (indirect) cost savings (for example, number of GP consultations) cannot be calculated from this study.

All analysis was performed using IBM SPSS Statistics (version 25) and SAS (version 9.4).

## Results

### Baseline characteristics

Twenty-two health centres with 117 GPs in the Utrecht region and four health centres with 41 GPs in the Rotterdam region participated in the study, with a corresponding total population of 195 394 patients (134 305 in the Utrecht region and 61 089 in the Rotterdam region) (Table 1). No significant differences in baseline characteristics were seen between health centres undergoing de-implementation strategy 1 and 2. None of the participating centres discontinued the intervention or were lost to follow up.

**Table 1.** Baseline characteristics of participating GPs, including pre-intervention numbers of vitamin testing

**Characteristic**	**Total**	**Intervention group**
Strategy 1(GP only)	Strategy 2(GP and patient)	*P* value
Health centres, *n*	26	13	13	
GPs,*n*	158	78	80	0.85
Male, *n* (%)	47 (30)	25 (32)	22 (28)	0.72
Population, *n*	195 394	97 658	97 736	0.99
SES, mean (range)^a^	0.26(−2.58–2.06)	0.53(−2.40–2.06)	0.21(−2.58–1.66)	0.48
Vitamin D tests pre-intervention, *n*	17 527	10 277	7250	0.30
Vitamin D/1000 patients pre-intervention, *n* (range)	88 (12–262)	102 (32–262)	75(12–150)	0.22
Vitamin B12 tests pre-intervention, *n*	12 304	7242	5062	0.36
Vitamin B12/1000 patients pre-intervention, *n* (range)	59 (7–198)^b^	69 (7–198)	49 (15–156)	0.21
GPs who followed e-learning, *n (%)*	76 (48)	28 (36)	48 (60)	0.11
GPs present at first training session, *n (%)*	50 (32)	26 (33)	24 (30)	0.86
GPs present at second training session, *n (%)*	50 (32)	24 (31)	26 (33)	0.85

^a^SES data, linked by four digital postal codes to location of health centre.^17^ ^b^Mean difference Utrecht-Rotterdam = –60 (95% CI = –94 to –26), *P* = 0.06. SES = socioeconomic status.

### Number of vitamin D and B12 tests

The total number of vitamin D tests ordered by GPs in strategy 1 and 2 decreased from 17 527 to 13 447 (–23%, OR 0.73, 95% CI = 0.71 to 0.75) with a range of –70% to +9%.

The mean number of vitamin D tests ordered in the pre-intervention year was 88/1000 patients (standard deviation [SD] 57), ranging per centre from 12 to 262/1000 patients. During the intervention year this was 66 vitamin D tests per 1000 patients (SD 51), resulting in a decrease of 22/1000 (95% CI = –32 to –13, *P*<0.001).

The total number of ordered vitamin D tests in non-participating health centres remained stable during the intervention year (–0.4%).

For vitamin B12 the total number of tests reduced from 12 304 (pre-intervention) to 9891 (–20%, OR 0.79, 95% CI = 0.76 to 0.81) with a range of –63% to +19%. Overall, a mean of 59 vitamin B12 tests/1000 patients (SD 43), was ordered during the pre-intervention year, compared to 47/1000 patients (SD 32) during the intervention year, resulting in a mean difference of 12/1000 (95% CI = –20 to –5, *P* = 0.003). The number of ordered vitamin B12 tests per 1000 patients per centre varied from 7 to 198/1000 patients during the pre-intervention year. A marked difference between the pre-intervention numbers for Utrecht (*n* = 46/1000 patients, SD 26) and Rotterdam (*n* = 106/1000 patients, SD 62) was found, which decreased to 38/1000 (SD 22) and 79/1000 (SD 43), respectively.

In non-participating health centres the number of tests decreased by 4.3%.

**Figure 2.**
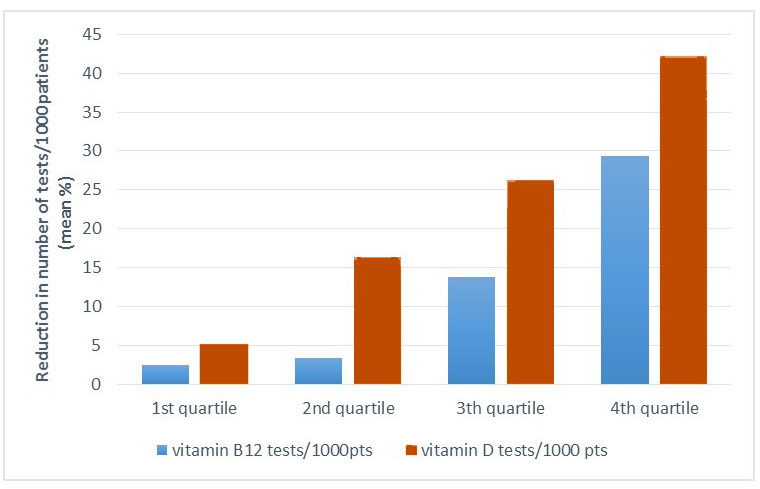
Number of ordered vitamin tests in pre-intervention period (in quartiles) related to reduction in number of ordered tests during intervention period. pts = patients.

All health centres were divided into quartiles according to the pre-intervention test ordering rates. For each category the mean reduction in ordered vitamin tests was calculated (Figure 2), which increased with higher pre-intervention test rates.

### Comparison between de-implementation strategy 1 and 2

In centres randomised to de-implementation strategy 1 (GP only) there was a 19% reduction in total number of vitamin D tests, compared with a reduction of 29% in centres randomised to de-implementation strategy 2 (GP and patient) (OR 0.88, 95% CI = 0.83 to 0.92) (Table 2).

**Table 2.** Reduction in number of vitamin D and B12 tests

**Characteristic**	**Total**	**De-implementation strategy**
Strategy 1 (GP only)	Strategy 2 (GP and patient)
Health centres, *n*	26	13	13
**Vitamin D**			
Reduction, % (range)	23(−9–70)	19(−4–70)	29(−9–28)
Absolute reduction in vitamin D/1000 patients, mean (range)	22 (−6–98)	24 (−6–98)	21 (−6–51)
**Vitamin B12**			
Reduction, % (range)	20(−19–63)	18(−3–63)	22(−19–42)
Absolute reduction in vitamin B12/1000 patients, mean (range)	12 (−6–69)	15 (−3–69)	9 (−6–63)

For vitamin B12 a reduction of 18% was found in centres of strategy 1 compared with a reduction of 22% in strategy 2 (OR 0.96, 95% CI = 0.91 to 1.02, non-significant) (Table 2).

### Vitamin test results

The mean test result of vitamin D tests did not differ before and during the intervention (56 nmol/L versus 55 nmol/L), neither for vitamin B12 (304 pmol/L). Also the proportion of test results below the reference values for vitamin D and B12 did not differ (around 17%, Table 3).

**Table 3.** Results of ordered vitamin D and B12 tests

	**Vitamin D**	**Vitamin B12**
Pre-intervention year	Intervention year	Pre-intervention year	Intervention year
Average value (range)	56 nmol/L (5–327)	55 nmol/L (10–309)	304 pmol/L(34–1476)	304 pmol/L(36–1476)
Female patients, %	72	72	72	72
Age, years, mean (SD)	48 (20)	47 (20)	51 (21)	50 (20)
Reference value: %	<50 nmol/L if >70 years: 30<30 nmol/L if <70 years: 17	<50 nmol/L if >70 years: 32<30 nmol/L if <70 years: 18	<148 pmol/l: 17	<148 pmol/L: 16

SD = standard deviation.

### Cost–benefit analysis

With an observed reduction of 4080 vitamin D and 2413 vitamin B12 tests, a saving of €32 640 and €14 719, respectively, can be calculated. Total savings were €47 359, compared with €20 340 related to development and implementation of the intervention (that is, development of patient education material [videos €12 566, booklets €1266, and posters €787], organisation of GP training sessions [€1096], and development of e-learning [€4625]). In case of an endurable implementation, only expenses for printing patient education material (€972), organisation of GP training sessions (€1096), and laboratory costs (€3000) for regular collection and (secured) communication of the number of test requests will continue, which is 11% of the total savings.

With an observed average reduction of 22 vitamin D and 12 vitamin B12 tests per 1000 patients, a cost reduction of €361 and €375, respectively, per standard primary care practice (2095 patients) per year can be calculated. In the Dutch context (with a total of around 5000 primary care practices) this would mean a total cost reduction of €3 681 701 per year; applying the 11% intervention costs results in total savings of €3 276 714 per year. This estimate is likely lower than the true savings because exact data on indirect cost reduction (for example, GP consultations) were not available for this analysis.

## Discussion

### Summary

This study demonstrates that with relatively simple and little time-consuming interventions the number of vitamin tests ordered in primary care can be reduced substantially. A 23% reduction in vitamin D tests and 20% in vitamin B12 tests ordered after 1 year was found, resulting in substantial cost savings.

Additional provision of patient information resulted in a 10% extra reduction of vitamin D tests on top of training and benchmarking of GPs, and a non-significant 4% additional reduction for vitamin B12 tests. The decrease was most prominent in centres that already had a high test ordering rate before the intervention.

### Strengths and limitations

A major strength of this study is the inclusion of a relatively high number of GPs. Furthermore, a simple and non-time-consuming intervention was used, which is easy to implement in daily practice.

However, some limitations need to be considered. First, primary care assistants were not included in the intervention although they sometimes issue laboratory forms on a patient request. Including primary care assistants might improve test reduction even more.

Second, benchmarking was provided at centre level, whereas provision of individual feedback could possibly result in further reduction of test ordering.

Furthermore, presence of at least one GP per centre was obligatory for participation in this study. It was not possible to check whether the lessons learned during the training sessions were actually shared with fellow GPs. However, an e-learning recording of the training sessions was available and 80% of all 158 GPs from the participating centres was reached via either the training session (32%) or e-learning (48%). Besides, participating GPs could have been influenced by other training programmes on the same topic during the intervention period, although no notifications of such programmes were received.

Selection bias may have occurred, because the GPs joining this study may have been more motivated compared with non-participating GPs. Also, regional differences were observed: Rotterdam region showed a much higher pre-intervention test rate and reduction in test ordering for vitamin B12. When interviewing participating GPs at the end of the intervention year, several Utrecht GPs mentioned that they experienced how the provided patient information raised awareness for vitamin testing, encouraging patients to ask for a vitamin B12 blood test.^8^ Reviewing the patient education material would therefore be required before further use.

Finally, reasons for testing were not studied during this project. It would have been interesting to know whether the GP training sessions helped to improve evidence-based testing behaviour. Presuming that the average test result is an adequate proxy for the quality of the indication of a performed test, the number of tests indicating a vitamin deficiency was determined, which did not differ during the intervention period.

### Comparison with existing literature

Several medical specialty societies have identified unnecessary laboratory testing as a target for overuse reduction in campaigns aimed at avoiding low-value care.^22,23^ Several studies focusing on vitamin D request reduction have been performed. Harnessing technology, such as redesigning electronic request forms,^14^ decision support tools,^15^ and obligatory addition of test indication on request forms^4^ showed reductions in vitamin D test requests of 36%, 67%, and 92%, respectively. Systematic reviews have also showed how performance feedback and clinician education,^24,25^ as well as patient education,^12,13^ are useful strategies with a solid evidence base for reducing use of low-value health services.

In the present study, the proportion of tests with a result below the reference values remained stable, suggesting that even in the intervention period many vitamin tests were still done without a valid indication. Several recent studies confirmed that a large proportion of vitamin D tests in primary care lack a valid clinical indication.^14,26,27^

### Implications for research and practice

In order to gain understanding of the sustainability of the present intervention, the authors looked at the number of vitamin D tests ordered in the 2 years following the pilot ‘practice improvement project’ performed in 11 primary care health centres (patient population 120 000) in Rotterdam. One year after the intervention the numbers remained stable; in the second year the number of vitamin D tests was only slightly higher (+0.3%, unpublished data). Because these results are based on a small project, further studies with a longer follow-up are necessary.

This study demonstrated that an intervention requiring limited time, consisting of training of GPs and benchmarking their diagnostic test ordering, can achieve a significant reduction in ordered laboratory vitamin tests. This strategy is most successful among GPs who frequently order vitamin tests and who are willing to improve their testing behaviour.

The low number of abnormal test results illustrates the need for training on evidence-based indications for vitamin test ordering. Training programmes and individual monitoring and feedback should be implemented on a national level to achieve a further reduction of the number of vitamin tests in primary care with substantial cost savings.
